# The usability and preliminary effectiveness of a web-based physical activity intervention in patients with knee and/or hip osteoarthritis

**DOI:** 10.1186/1472-6947-13-61

**Published:** 2013-05-28

**Authors:** Daniel Bossen, Cindy Veenhof, Joost Dekker, Dinny de Bakker

**Affiliations:** 1 Netherlands Institute for Health Services Research (NIVEL), P.O. Box 1568, 3500 BN, Utrecht, the Netherlands; 2 Department of Rehabilitation Medicine/EMGO, VU University Medical Center Amsterdam, Amsterdam, The Netherlands; 3 Department of Psychiatry, VU University Medical Center Amsterdam, Amsterdam, The Netherlands; 4 Tranzo, Tilburg University, Tilburg, The Netherlands

**Keywords:** Osteoarthritis, Physical activity, Web-based intervention, Development, Pilot study, Usability study

## Abstract

**Background:**

A large proportion of patients with knee and/or hip osteoarthritis (OA) do not meet the recommended levels of physical activity (PA). Therefore, we developed a web-based intervention that provides a tailored PA program for patients with knee and/or hip OA, entitled *Join2move*. The intervention incorporates core principles of the behaviour graded activity theory (BGA). The aim of this study was to investigate the preliminary effectiveness, feasibility and acceptability of *Join2move* in patients with knee and/or hip OA.

**Methods:**

A non-randomized pilot study was performed among patients with knee and/or hip OA. Primary outcomes were PA (SQUASH Questionnaire), physical function (HOOS and KOOS questionnaires) and self-perceived effect (7-point Likert scale). Baseline, 6 and 12 week follow-up data were collected via online questionnaires. To assess feasibility and acceptability, program usage (modules completed) and user satisfaction (SUS questionnaire) were measured as secondary outcomes. Participants from the pilot study were invited to be interviewed. The interviews focused on users’ experiences with *Join2move*. Besides the pilot study we performed two usability tests to determine the feasibility and acceptability of *Join2move*. In the first usability test, software experts evaluated the website from a list of usability concepts. In the second test, users were asked to verbalize thoughts during the execution of multiple tasks.

**Results:**

Twenty OA patients with knee and/or hip OA between 50 and 80 years of age participated in the pilot study. After six weeks, pain scores increased from 5.3 to 6.6 (p=0.04). After 12 weeks this difference disappeared (p=0.5). Overall, users were enthusiastic about *Join2move.* In particular, performing exercise at one's own pace without time or travel restrictions was cited as convenient. However, some minor flaws were observed. Users perceived some difficulties in completing the entire introduction module and rated the inability to edit and undo actions as annoying.

**Conclusions:**

This paper outlines the preliminary effectiveness, feasibility and acceptability of a web-based PA intervention. Preliminary results from the pilot study revealed that PA scores increased, although differences were not statistically significant. Interviews and usability tests suggest that the intervention is feasible and acceptable in promoting PA in patients with knee and/or hip OA. The intervention was easy to use and the satisfaction with the program was high.

**Trial registration:**

The Netherlands National Trial Register. Trial number: NTR2483

## Background

Osteoarthritis (OA) in the knee and hip is a degenerative joint disorder with a high prevalence that increases with age. The disease is associated with pain, functional disability and impaired quality of life [1,2]. OA is considered one of the major disabling diseases in the western world, affecting 10% of men and 18% of women over the age of 60 [3]. It has been recognized that regular physical activity (PA) is an effective lifestyle strategy in the management of OA [4-6]. However, to date the vast majority of OA patients remain sedentary [7-9]. In the long term, physical inactivity may lead to functional decline [10,11]. To maintain and improve physical function, the promotion of PA is a cornerstone in the treatment of OA [12].

Since general practitioners (GP) are considered the first and main point of contact for people with OA, the general practice is ideally situated to promote PA. In practice, however, a GP’s ability to encourage physical exercise is limited by time constraints and lack of standard protocols [13,14]. In particular, core elements concerning the risks of sedentary behaviour are insufficiently emphasized. At the same time, it is unlikely that OA patients will receive help elsewhere, since 90% are not referred to other health care professionals such as a physical therapist, orthopaedic doctor, rheumatologist or rheumatology trained nurse [15]. In this study, we call this group ‘outside-care patients’ and define them as those patients who did not have ‘face-to-face’ contact with a health care provider, other than a GP, for OA in the last six months.

The World Wide Web provides an alternative medium for reaching outside care patients. In Europe 61% and in North America 79% of the population have internet access [16]. Although the rate is lower in younger age groups [17], recent trends show that older people are among the fastest-growing internet users. To illustrate, in the Netherlands 95% of adults (55–65 years) and 75% of older adults (65–75 years) have access to internet in their home [18]. The internet is convenient, anonymous and appealing for those who want to work in their own environment and in their own time [19]. In particular, web-based interventions without the involvement of professionals have the potential to reach large populations, with a minimal burden on scarce health resources [20]. In recent years, several reviews reported that web-based interventions can be effective in promoting PA. Internet programs for patients with diabetes [21], multiple sclerosis [22] and heart failure [23] have led to the improvement of PA outcomes, even though effect sizes are small. Considering the potential of high reach and low costs [19], even these small effect sizes have large public health consequences. Given the advantages of the internet and its unique ability to reach outside care OA patients, we developed *Join2move.* Over the course of one year, we used an iterative design methodology to test, analyse and refine the *Join2move* program. As part of the iterative development process, this paper focuses on the preliminary effectiveness and the usability of *Join2move.*

### Join2move

Development was based on a systematic review [24] and a previously developed Behavioral Graded Activity (BGA) intervention [25]. The framework of the BGA program incorporates a baseline test, goal setting, time-contingent PA objectives (i.e. on fixed time points) and text messages to promote PA. An essential feature of the BGA program is the positive reinforcement of gradual PA, despite the presence of pain. The gradual increase in activities changes the perception that PA is related to pain and reinforces confidence to improve PA performance. This may lead to positive physical (e.g. physical capacity, muscle strength and joint mobility) and psychological changes (e.g. self-esteem, pain perception and anxiety). Due to the highly structured format of the BGA intervention, the internet constitutes a promising platform for translating BGA into a self-help format. The *Join2move* intervention is a fully-automated web-based intervention which contains automatic functions (automatic text messaging and automatic e-mails) without human support. Participants are initially presented with the homepage http://www.artroseinbeweging.nl. The password-secured PA program is available 24/7 from the homepage and is provided without charge. In keeping with the BGA treatment, the *Join2move* intervention is a self-paced nine week PA program in which patients’ favourite recreational activity is gradually increased in a time-contingent way. In the first week of the program, users select a central activity (e.g. cycling, walking or gardening), perform a 3-day self-test and determine a short term goal for the next eight weeks. Based on test performances and a short term goal, eight tailored weekly modules are automatically generated. Every week, new weekly assignments and evaluation forms (pain and performance) are posted on the password-secured website. If a scheduled weekly module is missed, users can choose to repeat the module, adapt the difficulty or continue with the next module. Since personal messages are updated on a weekly basis, users are encouraged to log in once a week. Automatic e-mails are generated if participants do not visit the website regularly. A description of the intervention is provided in Table 1.

**Table 1:** **Description of the *Join2move *intervention**

	
1. Filling out a PA Readiness Questionnaire (PARQ)	If participants answered “YES” to any of the seven PARQ, they were advised to see their GP before participation. If patients answered ‘NO’ to all of the questions, it was considered safe for them to engage in *Join2move.*
2. Provision of educational messages	Core elements of the program are presented on the personal website, including 1) focus on improving physical function rather than pain reduction; 2) first weeks can be accompanied by more pain;3) participant shares responsibility and has an active role.
3. Selection of a central PA	A favourite and a problematic activity are selected from an activity list, including walking, cycling, swimming etc.
4. Determination of baseline value via a 3-day self-test	To determine the baseline value, participants were requested to perform the selected activity three times a week until the pain threshold was reached. PA performances (minutes) and pain scores (1 to 10) were recorded in an online diary and stored on the website.
5. Setting a short and long term goal	In accordance with the baseline values, a range of goals is generated and presented on the website. Between the lower and upper limit of goals, patients could select a short term goal (9 weeks). Furthermore, a long term goal was set for 1 year.
6. Signing an agreement form	Participants sign an online agreement form. This form presents the short term goal and, again, core elements of the program.
7. Gradually increase selected activity (8 weekly modules)	Based on the short term goal, a tailored schedule of eight weekly modules is made on a time-contingent basis (i.e. fixed time points). The start of the schedule is slightly below the baseline value and increases incrementally towards the short term goal. Patients should not under-perform or over-perform this gradually increasing schedule. Every week, new modules and evaluation forms (pain and performance) are posted.

*GP* general practitioner, *PA* physical activity.

### Objectives

Extensive exploration is needed in order to examine the potential of the *Join2move* program. Consequently, our research question was:*“ What is the preliminary effectiveness (PA, physical function and self-perceived effect), feasibility and acceptability of Join2move in patients with knee and/or hip OA?”* “Feasibility” concerns whether we are capable of carrying out *Join2move* in a larger study. *“Acceptability”* is whether participants support or reject *Join2move.*

## Methods

### Pilot study

#### Study design and objective

This pilot study used a non-randomized design. Our primary focus was to determine the preliminary effectiveness of the *Join2move* intervention. A second purpose was to determine program use and user satisfaction with the *Join2move* intervention. This pilot study, which aimed to provide a basis for a large Randomized Controlled Trial (RCT), was part of a research protocol which has been approved by the ethics committee of the VU University Medical Center Amsterdam (Dutch Trial Register NTR2483).

#### Participants

Patients with self-reported knee and/or hip OA were recruited through advertisements in Dutch newspapers and online health-related websites. Eligibility criteria were 1) age 50–80; 2) self-reported OA in knee and/or hip; and 3) no physical therapy and/or treatment from a medical specialist for OA in the last six months. Potential participants were excluded if they 1) had no internet access at home, 2) were unable to understand the Dutch language and 3) had contra-indications (loss of consciousness and cardiovascular disease) for PA without medical supervision. To verify self-reported diagnosis, we performed clinical tests to assess the presence of knee and/or hip OA. Assessments were performed by a physiotherapist after the study period, according to the American College of Rheumatology (ACR) [26,27].

#### Procedure and measures

Interested patients who met the inclusion criteria were sent an invitation letter requesting informed consent. Once written informed consent was obtained, participants were invited to fill out a baseline questionnaire. After the baseline assessment, participants were assigned to the intervention. We conducted two online posttests at 6 and 12 weeks after baseline.

#### Preliminary effectiveness

To assess the potential effectiveness of the *Join2move* intervention, primary outcome measures in this study were PA, physical function and self-perceived effect. Secondary outcomes were OA symptoms, sport and recreation and quality of life. The first primary outcome, self-reported PA, was measured by the Short Questionnaire to Assess Health-enhancing PA (SQUASH) [28]. Pain scores and physical function were determined through a 10- point likert scale as well as the subscale pain of The Knee Osteoarthritis Outcome Score (KOOS) [29,30] and the Hip Injury Osteoarthritis Outcome Score (HOOS) [31,32]. The three secondary outcomes, symptoms, sport and recreation activity and quality of life, were also collected by using the HOOS and KOOS questionnaire. Descriptive statistics were used to analyse the data. Paired sample t-tests and regression analysis were used to determine the significance of the differences.

#### Feasibility and acceptability

To assess the feasibility and acceptability of the intervention, program usage and user satisfaction were measured as secondary outcomes. Program usage was measured by the number of weekly modules completed. Once a participant read the weekly assignments and filled out the evaluation form, the module was defined as completed. Adequate exposure to the program was achieved if users interacted at least 75% with the program content. This cut-off point was determined by the research team on the basis of previous research [33]. User satisfaction was measured via the System Usability Scale (SUS) [34]. Besides the usage and satisfaction, patients from the pilot study were invited for interviews to test user experiences. Semi-structured interviews were audio-recorded and transcribed with the interviewee's permission. An interview guide with open questions was employed to provide structure to the interviews. Transcribed texts were read and discussed to gain an overall understanding of the usability and user satisfaction.

### Usability tests

#### Participants

Two qualitative tests were performed to determine the usability of the *Join2move* intervention, viz.,1) heuristic evaluation, and 2) the Thinking Aloud approach. For the heuristic evaluation, four software experts from Netherlands Institute for Health Services Research (NIVEL) were invited to participate. With respect to the Thinking Aloud approach, five patients between the ages of 50–80 years with self-reported knee and/or hip OA were recruited via the Dutch Arthritis Foundation. The sample size for the Thinking Aloud approach was based on previous research by Nielsen [35]. The author claims that five users are enough to catch 85% of the usability problems.

#### Procedures and measures

The first usability test, the heuristic evaluation, was performed by means of a set of usability criteria created by Jakob Nielsen [36] and Dana Chisnell [37]. Nielsen [38] described heuristic evaluation as an informal method of usability testing that consists of a number of evaluators who are presented with an interface design and are then asked to comment on the errors and effectiveness of the product. Heuristics includes concepts such as “*Does the system behave consistently*?”, “*Does the site use words that older adults know?”, “Is the program perceived as helpful?”*(see Appendix 1 for the full list of heuristics). Software experts individually evaluated the website, based on the list of heuristics. Subsequent discussion yielded a list of usability issues. The second instrument, the Thinking Aloud approach [39], was used to consider how end-users interact with the intervention. In a home-based setting, test subjects were encouraged to verbalize their thoughts during the execution of multiple tasks. These tasks represented the major functionality of the intervention. Evaluations were carried out by two moderators. The procedure was video-recorded and transcribed afterwards.

## Results

### Pilot study

#### Participants

Of the 47 registered patients, fifteen (32%) did not meet the inclusion criteria. Reasons for exclusion were: no OA symptoms (n=3); receiving treatment from a physical therapist for OA (n=2); OA in other joints than knee or hip (n=7); and not meeting the age criteria of 50–80 years (n=3). Furthermore, seven (15%) participants did not return the informed consent document and five (11%) participants withdrew after returning informed consent. A total of twenty (42%) participants were finally included. Sixteen (80%) participants agreed to be interviewed. According to the ACR criteria, thirteen of the sixteen participants (81%) had clinical knee and/or hip OA, and three participants (19%) had no OA. Participants’ demographic characteristics are shown in Table 2.

**Table 2:** Demographic and clinical characteristics

**Participants (N, %)**		
Gender		
Male	5	25
Female	15	75
Age (years, SD)	64	6.6
Location OA (N, %)		
Knee	7	35
Hip	5	25
Knee and hip	8	40
Duration OA symptoms (years, SD)	9.3	11.4

OA, osteoarthritis; SD, standard deviation.

#### Preliminary effectiveness

PA results at baseline, six weeks and twelve weeks are given in Table 3. Over the twelve week period, the total time spent on PA increased from 1,697 to 2,044 min/week, and the time spent on moderate intensity increased from 323 to 553 minutes a week. These results, did not however, attain statistical significance (p= 0.3 and p=0.43, respectively). At 6 weeks, patients did report significantly higher levels of pain compared to the baseline - from 5.3 to 6.6 (p=0.04). After twelve weeks the differences were no longer statistically significant (p=0.5). With regard to physical function, a small, non-significant increase was observed (Table 4).

**Table 3:** Comparison of change in PA levels (mean and SD)

**PA (mean, SD)**	**Baseline**	**Baseline**	**Baseline**
	**(n=20)**	**(n=20)**	**(n=20)**
Total PA (min)	1697 (1174)	2108 (1206)	2044 (1369)
Moderate PA (min)	323 (330)	539 (549)	553 (673)
Pain (0–10)	5.3 (1.7)	6.6 (2.0)*	5.2 (1.8)

*p<0.05 compared with baseline. *PA* Physical Activity. For (moderate) PA a higher score indicates an improvement. For pain, a lower score indicates an improvement.

**Table 4:** HOOS and KOOS scores (mean and SD)

	**HOOS**	**HOOS**	**HOOS**	**KOOS**	**KOOS**	**KOOS**
	**baseline**	**6 weeks**	**12 weeks**	**Baseline**	**6 weeks**	**12 weeks**
Pain (0–100)	54.2 (19.2)	55 (16.0)	59.3 (17.1)	45.6 (18.5)	47.8 (17.4)	49.1 (15.1)
Symptoms (0–100)	49.6 (16.5)	48.9 (13.7)	58.8 (16.2)	61 (16.8)	55.2* (16.0)	62.6 (14.9)
ADL (0–100)	53.2 (20.3)	49.2 (14.9)	54.9 (17.4)	46.8 (20.1)	46 (14.9)	47.5 (20.6)
Sport (0–100)	33.3 (23.4)	18.8* (18.0)	45.1 (33.9)	18.2 (16.1)	16.3 (18.6)	15 (19.1)
QOL (0–100)	37 (18.8)	38.5 (13.7)	41 (12.9)	27.9 (17.7)	32.9 (14.1)	34.1 (12.0)

p<0.05; HOOS/KOOS, The Hip/Knee Osteoarthritis Outcome Score; ADL, activities of daily life; QOL, Quality of life. For all outcomes a higher score indicates an improvement.

#### Feasibility and acceptability

The majority of participants (n=12, 60%) selected walking as the central activity. Other selected activities were floor exercises (n=3, 15%), cycling (n=1, 5%), domestic tasks (n=1, 5%), gardening (n=1, 5%), and rowing (n=1, 5%). A total of twenty participants commenced the intervention with the program introduction. Login-file analyses revealed that 100% (n=20) of the users completed the introduction module. Overall, 55% (n=11) of the participants completed at least 75% of the program (≥7 week assignments). 70% (n=14) achieved 60% program exposure and 30% (n=6) were exposed to at least 30% of the intervention. The exposure percentage declined over time. The most listed reasons for skipping a weekly PA were other commitments or of lack of time. Adverse events, such as extreme pain or injuries, were not reported during the program. The 16 interviews revealed that performing the activities in one's own time and at one's own pace was regarded as convenient. In general, participants perceived the website as an additional motivation to perform PA. However, the interviews also revealed an important usability issue. It became clear that patients were dissatisfied with the rigid character of *Join2move.* As one user commented “*When I skipped my weekly PA exercise due to other commitments, I had no opportunity to repeat that exercise. That was frustrating”.* The results from the SUS among 15 participants revealed an average score of 73 points (SD 15) on a 100-point scale questionnaire. According to the study of Bangor et al. [40], this score can be considered “good”. Only two patients disagreed with the statement “*The website was easy to use*” and nearly all patients disagreed with the statement *“I think I would need technical support to be able to use the program”.*

### Usability tests

Experts in heuristic evaluation rated the rigid character of the intervention as a disadvantage. This was in accordance with results from the interviews. Results of the Thinking Aloud test are given in Table 5. The majority of tasks were completed as expected. Of the 15 tasks presented, on average, 12 (80%) were completed successfully. However, several usability problems were identified. Respondents had difficulties in logging (task 4), completing the introduction module (task 5) and establishing their personal starting level (task 14). On all occasions, navigation to Aim of the Program (task 10) was not executable due to an error in the system.

**Table 5:** Thinking Aloud test among 5 participants

**Tasks**	**Average time (sec)**	**Task correct**
1) What is the moderator's telephone number?	15.60	100% (n=5)
2) Register yourself for the program	308.40	100% (n=5)
3) Search for information about healthy weight and osteoarthritis	68.60	80% (n=4)
4) Login (with your username and password)	85.60	60% (n=3)
5) Complete module 1 (introduction)	352.40	40% (n=2)
6) Navigate to the webpage ‘Symptoms’	48.20	60% (n=3)
7) Navigate to the webpage ‘My profile’	12.20	100% (n=5)
8) Watch home exercise video No. 4	23.00	80% (n=4)
9) Write something in your workbook	84.60	100% (n=5)
10) Navigate to the webpage ‘Programme Aim ”	82.80	0% (n=0)
11) Log out	2.00	100% (n=5)
12) Log in, once again.	59.40	100% (n=5)
13) Fill in the evaluation form (performance and perceived pain)	62.00	100% (n=5)
14) Check the starting point of your programme in minutes	73.80	60% (n=3)
15) Check your most recent update in your workbook	16.20	100% (n=5)

#### Adjustments

Based on the results of the interviews and the heuristic evaluation, we changed the program's time contingent structure (i.e. fixed time periods) into a more flexible format. In the most recent version, options have been included which give users the choice of repeating modules and adapting the difficulty of the modules. The usability errors from the Thinking Aloud approach had more to do with the design of the website and the location of several buttons. These relatively minor problems were also addressed.

## Discussion

Results from this study indicate that *Join2move* is a plausible, feasible and acceptable program for patients with knee and/or hip OA. Although effectiveness was not proved due to the lack of power, results do indicate that *Join2move* has the potential to increase PA levels in patients with knee and/or hip OA. Participants reported higher levels of PA, particularly (and as expected) involving moderate activities like walking and cycling (200 minutes). In line with other research [41], walking was by far the most frequently selected activity. Our positive results correspond with a comparable face-to-face intervention, showing a moderate PA increase of 170 minutes [25]. In the first three weeks, the increase was accompanied by more pain. Fortunately, after twelve weeks the pain scores declined towards baseline levels. Although the intervention focused on improving PA rather than on pain reduction, the increased pain was certainly a reason for concern. The precise cause of observed elevated pain scores is unclear. A possible explanation is the increased PA which may generate more muscle and joint pain. However, it is important to note that higher levels of pain are not associated with deterioration of OA [42,43].

Providing an intervention does not automatically mean that patients will use it, particularly when it is self-directed, with minimum personal contact. Since the success of web-based interventions requires active participation, non-usage attrition has been pointed out as a common concern in the field of web-based education. In line with other studies, [21,22,44], the number of users gradually decreased during the nine-week program. Overall, 55% (n=11) of the participants completed at least 75% of the program. This exposure percentage corresponds with the study of Steele et al. [33] and can be rated as reasonably high for web-based interventions without human interference. The delivery of personal information on a weekly basis is a possible explanation for this relatively low non-usage attrition. In this respect, it was not possible for users to run the entire program at one time. Although we did not examine the specific strategies of engagement, the authors assume that the week-by-week basis provided an incentive to return to the website.

With respect to usability, the involvement of end-users was extremely valuable for identifying usability issues and system flaws. Along the way, we incorporated greater flexibility into the program. The implemented changes resulted in a less rigid version with more options tailored to the performance of the individual user.

The findings from this study need to be interpreted in light of the study's limitations. The small sample size, single group design and lack of long-term assessments limit conclusions of causality, long-term effects and generalizability. Furthermore, the potential presence of the so-called Hawthorne effect may have contributed to an overestimation of PA scores. This implies that observed PA changes may be partly the result of study participation. Besides the Hawthorne effect, self-reported PA measures may also contribute to an over-estimation of PA levels in this study. This may be a consequence of recall error, perceived social desirability and other biases. To obtain the best results, a combination of validated questionnaires with objective measures would be preferable in future studies. Another limitation concerns outside-care patients who lack computer skills or internet access. These groups are mostly excluded from web-based interventions. Unfortunately, this disadvantage applies also to the *Join2move* intervention. Typically, these patients are disproportionately less educated and have a lower income. Particularly with regard to these under-served populations, GPs should refer sedentary OA patients more frequently to a physical therapist or other health care provider. Further, it will be important to translate *Join2move* for other self-help platforms, such as videos, brochures and self-help books. A final limitation is that we only performed one Thinking Aloud test to detect and resolve usability issues. Unfortunately, we did not retest the redesigned intervention. In order to optimize usability for the implementation phase, a repetition of this procedure is advised.

## Conclusions

Strong evidence indicates that regular PA is important in the management of OA. To date, however, many patients with knee and/or hip OA remain sedentary. Unfortunately, the vast majority of these patients do not receive any help in the promotion of PA. Low-cost, effective and accessible PA interventions are needed. Although our results are not conclusive, this study suggests that *Join2move* has the potential to contribute to meeting this need. The intervention is unique, since this is the first web-based PA intervention focusing on outside-care patients with OA. Moreover, while most web-based PA interventions have additional human contact, the *Join2move* intervention is fully computerized. Given the fully automatic character, the program has the potential to reach large populations while placing a minimal burden on our scarce health resources. This paper illustrates how involving end-users and experts can contribute successfully to the development of a web-based self-help intervention. The results suggest that the intervention is feasible and acceptable in promoting PA among patients with knee and/or hip OA. The intervention was easy to use and satisfaction with the program was high. This suggests that the intervention is acceptable for patients with knee and hip OA. Preliminary results from the pilot study revealed that PA scores increased, although differences were not statistically significant. A randomized controlled trial is needed to determine the effectiveness of the *Join2move* program.

## Appendix 1

Usability items used for the heuristic evaluation

Interaction

1) Are the links to websites consistent throughout the website?

2) Do buttons and links show that they have been clicked?

3) Does the ‘back’ button appear on the browser toolbar on every page?

4) Are error pages descriptive, and did they provide a solution to the user?

5) Does the system inform users what is going on through appropriate feedback within a reasonable time frame?

6) Does the system behave consistently?

7) Does the system eliminate error-prone conditions and present users with confirmation options before they commit to the action?

Information and architecture

8) Is the path for any given task a reasonable length (2–5 clicks)?

Visual design

9) Is the default font size 12-point or larger? If not, is there an obvious way on the page to increase the font size? If not, does changing the font size in the browser enlarge all of the text?

10) Are text and interaction elements a different colour from the background? Are clickable items highlighted differently from other non-clickable highlighted items?

Information design

11) Has the amount of text been minimized; is only necessary information presented?

12) Is the content written in the active voice, directed to “you”?

13) Does the site use words that most older adults know? Are instructions written in plain language?

14) Is a relevant help button provided? Does the system provide documentation about the website?

Persuasive principles

15) Can users relate to and feel familiar with the context, images and figures that appear in the program?

16) Does the system contain the knowledge to be learned?

17) Is the program easy to use and are the tasks easy to perform with a small number of steps and keystrokes?

18) Can users learn about how they solved the tasks on previous occasions when the system was used?

19) Are users aware that the moderator can observe and see the results?

20) Do users get rewards or praise when a task is performed correctly

21) Is the program perceived as helpful?

22) Does the program act as a coach?

## Competing interests

The authors declare that they have no competing interests.

## Authors’ contributions

DB was responsible for day to day management of the project, developed the web-based intervention, collected all data, analysed the data and wrote the paper. CV had the idea for the study and managed the project. JD and DdB contributed to the intellectual content of the manuscript. All authors read and approved the final document.

## Pre-publication history

The pre-publication history for this paper can be accessed here:

http://www.biomedcentral.com/1472-6947/13/61/prepub
